# Stability of response characteristics of a Delphi panel: application of bootstrap data expansion

**DOI:** 10.1186/1471-2288-5-37

**Published:** 2005-12-01

**Authors:** Ralitsa B Akins, Homer Tolson, Bryan R Cole

**Affiliations:** 1 Quality and Patient Safety Initiatives, Rural and Community Health Institute, The Texas A&M University System Health Science Center, College Station, Texas, USA; 2 Department of Educational Administration and Human Resource Development, The Texas A&M University, College Station, Texas, USA

## Abstract

**Background:**

Delphi surveys with panels of experts in a particular area of interest have been widely utilized in the fields of clinical medicine, nursing practice, medical education and healthcare services. Despite this wide applicability of the Delphi methodology, there is no clear identification of what constitutes a sufficient number of Delphi survey participants to ensure stability of results.

**Methods:**

The study analyzed the response characteristics from the first round of a Delphi survey conducted with 23 experts in healthcare quality and patient safety. The panel members had similar training and subject matter understanding of the Malcolm Baldrige Criteria for Performance Excellence in Healthcare. The raw data from the first round sampling, which usually contains the largest diversity of responses, were augmented via bootstrap sampling to obtain computer-generated results for two larger samples obtained by sampling with replacement. Response characteristics (mean, trimmed mean, standard deviation and 95% confidence intervals) for 54 survey items were compared for the responses of the 23 actual study participants and two computer-generated samples of 1000 and 2000 resampling iterations.

**Results:**

The results from this study indicate that the response characteristics of a small expert panel in a well-defined knowledge area are stable in light of augmented sampling.

**Conclusion:**

Panels of similarly trained experts (who possess a general understanding in the field of interest) provide effective and reliable utilization of a small sample from a limited number of experts in a field of study to develop reliable criteria that inform judgment and support effective decision-making.

## Background

Since its development in the 1950's, the Delphi method has been broadly utilized in various fields of study including clinical medicine, nursing practice, medical education and healthcare services [1,2]. Despite its wide application, however, many questions regarding this methodology still continue to intrigue researchers. One such question is whether small expert samples are sufficient to conduct a Delphi study; *sufficient *in this case refers to the stability of panel responses.

In this methodological paper, the adequacy of utilization of a small number of experts in a Delphi panel is discussed. Computer software (SPSS 12.5) was used to augment the data from the first round of a Delphi survey conducted with 23 healthcare quality and patient safety experts and to study the similarities and differences between the response characteristics of the original data and the computer-generated samples.

### Sample size in delphi studies

There is no agreement on the panel size for Delphi studies, nor recommendation or unequivocal definition of "small" or "large" samples [1-3]. There is a lack of agreement around the expert sample size and no criteria against which a sample size choice could be judged. Studies have been conducted with virtually any panel size. Reid (1988) studied published articles on Delphi applications in healthcare and noted that there were from 10 to 1685 panellists utilized [4]. Delphi studies with fewer than 10 participants are rarely conducted. For example, a panel of only 5 experts was asked to identify serious drug interactions most likely to occur in the ambulatory pharmacy setting [5], and the responses of an international panel of 6 experts were used to explore competence training for primary care nurses [6]. Many published Delphi studies use panels consisting of 10 to 100 or more panellists, as demonstrated by the following examples. A panel of 10 experts evaluated stage-tailored health promoting interventions [7], and 13 experts were utilized in studying a variety of skills in young children [8]. Two expert panels, consisting of 18 regional and 52 national experts respectively, participated in evaluating an existing pain evaluation system [9]. A multidisciplinary group of 23 participants developed recommendations for the treatment of gastroesophageal reflux disease [10]. Thirty participants were utilized to examine the factors impacting the effectiveness of continuing education in long-term healthcare environment [11] and 32 experts identified the types of scientific misconduct most likely to influence the results of a clinical trial, such as selective reporting and opportunistic use of the play of chance [12]. Two separate studies used a panel of 60 individuals to explore issues of nurse leadership in primary care and priorities in cancer nursing research, respectively [13,14]. Sixty-four medical educators participated in a Delphi panel to develop guidelines for bioterrorism curricula for medical students [15]. One hundred and ten panellists participated in identification of health areas with consumer involvement in research [16], and another panel of 110 pharmacists identified challenges for pharmacy executives [17]. A Delphi panel of 199 nurses explored paediatric oncology nurses' perceptions of parent educational needs [18]. The University of Virginia used 421 respondents in one Delphi study [19]. In another Delphi study, 2,865 participants were invited to participate and 1,142 returned their questionnaires [20].

The sample size in Delphi studies has been researcher and situation specific, and more often than not, convenience samples have been chosen dependent on availability of experts and resources. Given the lack of any Delphi sample size standards, there is confusion regarding how small a "small" sample can be. For example, in one Delphi study, a sample of 37 participants was considered too small for a definite conclusion [21]. In general, the confusion around the Delphi sample arises from the fact that there are no standards established in any methodologically acceptable way. The current literature presents only empirical choices on Delphi expert sample sizes made by individual researchers, such as convenience, purposive or criterion sampling [22].

### Malcolm Baldrige award criteria for performance excellence in healthcare

The Malcolm Baldrige National Quality Award (MBNQA) was established in 1987 and is the most prestigious national quality award in the U.S. It is given by the United States Department of Commerce under the authority of the Malcolm Baldrige National Quality Improvement Act of 1987. The MBNQA recognizes superior continuous improvement programs focused on achieving and sustaining performance excellence for the long term.

The MBNQA framework consists of core values and concepts embodied in seven criteria categories: (1) Leadership; (2) Strategic planning; (3) Focus on patients, other customers and markets; (4) Measurement, analysis and knowledge management; (5) Staff focus; (6) Process management; and (7) Organizational performance results [25]. Although the criteria are results-oriented, they are non-prescriptive and adaptable, so that organizational structure and quality approaches may differ widely from one organization to another. The flexibility and adaptability of the Baldrige framework allow improvement changes and objective assessment of an organization's quest for quality.

The MBNQA has five sector categories: Manufacturing, Service, Small Business, Education, and Healthcare. The category of healthcare was added in 1998. Eligible applicants in the category of healthcare include hospitals, health maintenance organizations, long-term healthcare facilities, healthcare practitioner offices, home health agencies, and dialysis and ambulatory surgery centers.

Healthcare experts familiar with the MBNQA approaches are best suited to offer a systems approach. Therefore, their similar training, knowledge and understanding were targeted influences for the outcomes of the Delphi survey. The Malcolm Baldrige criteria provided a structured approach for producing results within a proven framework. Knowledge of the intricate details of the framework ensured an advantage in its application to the field of patient safety.

### Importance of the study findings

The results from the analysis of and the comparison between the original responses of the expert panel and the computer generated samples indicated that the number of selected experts utilized in this panel was sufficient to insure reliability for a Delphi study in the field of interest. This finding is important because it establishes the stability of the results from a Delphi survey conducted with a small number of experts from a defined field of study. We can hypothesize that Delphi surveys with a similar number of experts with similar training and knowledge in other fields of study would also yield stable results. Additionally, this finding is important for practitioners in the field of quality training, showing that individuals with similar training, knowledge and understanding of the systems approach based on the Malcolm Baldrige quality criteria could be utilized in a Delphi panel with a constricted number of experts. Given the fact that specialized experts in a given field may be limited, the results of this study suggest that utilization of a small expert sample from limited numbers of experts in a field of study may be used with confidence.

## Methods

### The Delphi method

The Delphi method facilitates communication between and among a panel of experts, so that the process is effective and the group as a whole can deal with a complex problem [26]. This method improves the generation of critical ideas by structured collection of information and processing of the collective input from a panel of geographically dispersed experts [27]. The methodology originated in the early 1950's, when an Air Force-sponsored Rand project, titled "Project Delphi" sought to reach consensus, through a series of questionnaires and controlled feedback, among military experts on possible U.S. industrial targets for attacks from Russia [26]. The Delphi methodology has applications in many fields, including healthcare, education and sociology.

The advantages of the method are numerous and include:

• The ability to conduct a study in geographically dispersed locations without physically bringing the respondents together;

• Time and cost-effectiveness;

• Discussion of broad and complex problems;

• The ability for a group of experts with no prior history of communication with one another to effectively discuss a problem as a group;

• Participants can have sufficient time to synthesize their ideas;

• Participants can respond at their convenience;

• There is a record of the group activity that can be further reviewed;

• The anonymity of participants provides them with the opportunity to freely express opinions and positions;

• The process has proven to be effective in a variety of fields, problems, and situations [28].

Researchers use the Delphi method to translate scientific knowledge and professional experience into informed judgment, and support effective decision-making [22]. For subject matters in which the best available information is the judgment of knowledgeable individuals, the Delphi method has demonstrated decision-making advantages over traditional conferences, group discussions, brainstorming, and other interactive group activities. The focus in a Delphi study is on the stability of the group opinion rather than on individuals' opinions, thus measuring the group result is superior to measuring individual rankings [27].

### Healthcare Delphi survey

A Delphi survey with 23 experts from 18 US states was conducted to create a patient safety tool to guide patient safety improvement in US healthcare organizations. The MBNQA framework was used as a general matrix for the tool and was extended to the field of patient safety. The Delphi study was reviewed and approved by the Institutional Review Board – Human Subjects in Research at Texas A&M University (protocol # 2003–0071).

The MBNQA examiners are trained to have in-depth knowledge and extensive experience relevant to the seven Baldrige categories in at least one, and preferably more than one industry or service sector. Consequently, it was important that the Delphi panel members had expertise in the application of the Baldrige process, as well as in patient safety systems.

### Study sample size selection

Given that the intent of the Delphi survey was to examine the patient safety systems in the context of a nationally accepted management framework (the Malcolm Baldrige National Quality Award Criteria for Performance Excellence in Healthcare), all study experts were selected using stringent criteria, including knowledge of and/or training in the Malcolm Baldrige Criteria for Performance Excellence in Healthcare, and knowledge and experience in patient safety. The number of experts with such qualifications was fairly limited (n ~ 100) and the sample of Delphi panel participants was small (n = 23).

The sample size for the study was based initially on an empirically selected small sample size (n = 15) and the expected response rate necessary to achieve this sample size. It was critical to consider what response rate was usually obtained in surveys in the particular study area (healthcare quality and patient safety). A survey on the quality of healthcare and the problem of medical errors administered to a large random sample of Colorado physicians, national physicians and Colorado households, revealed response rates of 66% for the Colorado physician sample, 36% for the national physician sample, and 82% for the Colorado household sample [23]. The psychometric validation process for the Safety Attitude Questionnaire conducted in 160 healthcare sites in the U.S., England and New Zealand obtained a response rate of 67% [24]. Sumsion (1998), as discussed by Hasson, Keeney and McKenna (2000), argued that in order to maintain the rigor of the Delphi technique, a response rate of 70% must be maintained [22]. Based on the healthcare study response rates as found in the literature, it was concluded that for this study a response rate of 70% could be expected. Thus, to obtain at least 15 respondents, the study should begin with 22–23 Delphi panellists, where a sample size of 15 to 23 respondents was considered to be small. Responses were obtained from all 23 experts that had made a commitment to serve on the Delphi panel.

### Selection of Delphi experts

Delphi participants are not selected randomly; rather, they are purposefully selected to apply their knowledge and experience to a certain issue based on criteria, which are developed from the nature of the problem under investigation. The following criteria were utilized to qualify experts in healthcare quality improvement and patient safety for inclusion in the original Delphi panel:

(a) Judges, senior examiners or examiners for the Malcolm Baldrige National Quality Award in healthcare;

(b) Senior administrators in healthcare institutions that have won or have applied for the Malcolm Baldrige National Quality Award in healthcare;

(c) Senior administrators in healthcare institutions that have won a state quality award within the last five calendar years;

(d) Leaders in state or national organizations or programs that emphasize continuous quality improvement and/or patient safety;

(e) Experts possessing more than one of the aforementioned criteria.

Based on these criteria, only about 100 healthcare experts nationwide qualified for participation in the Delphi panel. Barriers to identification and inclusion of experts were the confidentiality of MBNQA applicant names and the scarcity of healthcare quality award winners at a state level. Approximately one quarter of the qualified experts were recruited for participation in the panel.

Since the names of the healthcare institutions, which have applied for the Malcolm Baldrige Award are kept confidential, obtaining information regarding the application status of a healthcare institution is a subject of individual contacts and institution's willingness to share such information. The reviewers for the category of healthcare available through the *Malcolm Baldrige list of examiners *were reached via phone and asked if they would consider sharing information on the applicant status of their institutions. Information was also solicited whether the examiners' organizations had won state quality awards within the last five years, and whether the examiners were senior administrators in their respective institutions. If the examiners and senior healthcare administrators qualified as experts in healthcare quality improvement and patient safety according to the study criteria described above, they were invited to participate in the study. In general, the study participants were recruited via telephone and/or letter contact and were selected from (1) the list of Malcolm Baldrige examiners, (2) senior administrators from healthcare institutions that had won national and/or state quality awards, and (3) referrals from (1) and (2). The recruitment of participants was discontinued after 23 qualified individuals confirmed their willingness to serve on the Delphi panel.

### Importance rating scale

The Delphi panel utilized a four-choice Likert scale for assessing the importance of suggested critical processes for patient safety systems in healthcare institutions. The scale was modelled according to the original importance scale developed by Turoff [26]. The participants in the panel were asked to indicate the importance of the Delphi survey items from 1 to 4, where "4" represented processes *very important *to patient safety systems in healthcare institutions, and "1" represented *unimportant *(irrelevant). All survey items that were identified by the expert group as "very important" or "important" for patient safety in the third study round, when the experts reached consensus, were included in the final patient safety tool. The Delphi survey concluded in three rounds with creation of a process-centred tool for evaluating patient safety performance and guiding strategic improvement at the institutional level, extending the MBNQA criteria to the area of patient safety [29].

### Bootstrap study design

After the Delphi panel created the patient safety tool, the concern about possible group bias with small expert numbers remained, because it has been argued that increased group size is beneficial in Delphi surveys [27]. To study possible differences in response characteristics and to explore the possibility for group bias in the study group of experts and, therefore, to assess the possible error in the creation of the patient safety tool, we generated via computer program (SPSS 12.5) two large samples of expert ratings. Since the variation in expert opinions was greatest in the first study round, encompassing the whole spectrum of possible ratings from 1 to 4, the results from the first survey round were utilized as the basis for computer generation of the expanded samples. The expert responses to the survey items were randomly selected *with replacement *by the computer program based on the raw data from the first round for the actual survey experts. This resampling technique is called *bootstrap*.

The bootstrap method was developed by Efron in 1979 and has found wide use in the field of applied statistics [30]. Bootstrap is a Monte Carlo-type data augmentation method utilizing resampling with replacement that can be used with observed data. While Monte Carlo techniques usually generate fictitious data, bootstrap resamples with replacement from the original observed values and generates multiple bootstrap samples as a proxy to the independent real sample. Each bootstrap sample is a random sub-sample (with the same size as the original sample) taken with replacement from the observed values. The original sample is treated as the "virtual population" and the sample is duplicated multiple times. The procedure can be repeated as many times as desired. Resampling has proven valid for any kind of data, including random and non-random data [31]. During the last three decades, the bootstrap resampling has been used widely in applied statistics [32].

### Advantages and limitations of the bootstrap technique

Resampling (bootstrapping) of a random sample of an unknown population is considered to model the distribution of that population, where the vaguer the knowledge about the population distribution is, the more valuable the bootstrapping technique proves to be [33]. Since classical statistical techniques are primarily designed for parametric statistics with normal distributions, the bootstrap technique has an advantage in distributions with no convenient statistical formulae, overcoming the limitations of the classical approaches in working with small sample sizes and non-normal distributions [34]. Efron and Tibshirani proposed that the technique reduces the assumptions required to validate analysis and eliminates theoretical calculations required to assess accuracy; its major application is in determination of confidence intervals, where 1,000 or more iterations are necessary to estimate the confidence intervals [30]. The simplicity of the method allows its application in a wide variety of studies and is considered superior to standard statistical tests of significance because it reduces the threat of multiple comparisons bias and provides information on the distribution of scores (and not parametric distributions); the technique is not dependent on a specific nominal size such as 5% and therefore is more accurate [34,35]. The bootstrap technique may have limited accuracy in very small sample sizes (n < 20), in extremely skewed distributions, and if extreme outliers are present [34].

### Data manipulation

In this study, statistics for each bootstrap resample were saved in memory and later used for estimation of sampling variance, confidence intervals and assessment of bias for the raw data [30]. The characteristics of the generated samples, when analyzed collectively are used to provide a more representative expression of the underlying population, in this study – the population of patient safety experts knowledgeable about the Malcolm Baldrige framework. The *hypothesis *was that strict expert inclusion criteria based on training in, and knowledge and understanding of the MBNQA framework in the original sample of 23 experts would provide stability of responses, even if the number of responses was increased by computer generated bootstrap samples.

The bootstrap samples were generated using SPSS 12.5 software. The characteristics of the SPSS 12.5 model were as follows:

 NonLinear Regression

 MODEL PROGRAM b0 = 2

 COMPUTE PRED_ = b0

 CNLR VAR00001

 /OUTFILE='C:\DOCUME~1\User\LOCALS~1\Temp\spss452\SPSSFNLR.TMP'

 /PRED PRED_

 /BOOTSTRAP 1000 [2000]

 /CRITERIA STEPLIMIT 2 ISTEP 1E+20.

More specifically, the *regression *routine and the subroutine of *nonlinear regression *were employed. Once these routines were selected, the *options *feature of nonlinear regression was invoked. The *bootstrap *option was selected and the "paste" option was used to indicate the number of bootstrap samples to be derived.

## Results

The patient safety tool created by the Delphi panel was based on the seven Malcolm Baldrige categories and included 54 survey items (38 critical processes ranked directly, and one indirectly defined by 16 performance measures). For each survey item, the following inferential statistics were derived:

- *Mean *(average) – the measure that represents the arithmetic average for the group of experts

- *95% confidence interval *– representing the upper and lower limits between which 95% of the sample expert scores will be expected to fall

- *5% trimmed mean *– calculation of experts' average score with exclusion of the highest and lowest 5% of the scores; the difference between the mean and the trimmed mean shows whether there are many outliers in the rankings among the experts in the sample (real or computer generated)

- *Standard deviation *– describes the variability of the score distribution.

These descriptive statistics were calculated for each of the following three samples:

(a) original expert responses of 23 experts in healthcare quality and patient safety (n = 23)

(b) augmented-computer-generated sample of 1000 iterations of resampling and the original sample (n = 1001)

(c) augmented-computer-generated sample of 2000 iterations of resampling and the original sample (n = 2001).

The results for each of the calculated statistics (for each of the three groups of data) are presented in Table 1.

**Table 1:** Comparison between statistical data for the original and augmented samples

**Survey Item**	**Data Entries**	**Mean**	**95% CI**	**5% Trim. Mean**	**SD**
1	Participants, n = 23	3.64	3.39–3.88	3.70	.5680
	Iterations, n = 1001	3.64	3.63–3.65	3.64	.1157
	Iterations, n = 2001	3.64	3.63–3.64	3.64	.1172
2	Participants, n = 23	3.64	3.39–3.88	3.70	.5680
	Iterations, n = 1001	3.64	3.63–3.65	3.64	.1154
	Iterations, n = 2001	3.64	3.63–3.64	3.64	.1151
3	Participants, n = 23	3.46	3.12–3.79	3.55	.7820
	Iterations, n = 1001	3.45	3.44–3.46	3.46	.1582
	Iterations, n = 2001	3.46	3.45–3.46	3.46	.1589
4	Participants, n = 23	3.26	2.99–3.53	3.29	.6190
	Iterations, n = 1001	3.26	3.26–3.27	3.27	.1257
	Iterations, n = 2001	3.26	3.25–3.26	3.26	.1276
5	Participants, n = 23	3.13	2.89–3.37	3.14	.5480
	Iterations, n = 1001	3.13	3.13–3.14	3.13	.1093
	Iterations, n = 2001	3.13	3.13–3.14	3.13	.1133
6	Participants, n = 23	3.22	2.93–3.51	3.24	.6710
	Iterations, n = 1001	3.22	3.21–3.23	3.22	.1347
	Iterations, n = 2001	3.22	3.21–3.22	3.22	.1371
7	Participants, n = 23	3.11	2.85–3.36	3.12	.5950
	Iterations, n = 1001	3.10	3.09–3.11	3.10	.1235
	Iterations, n = 2001	3.10	3.10–3.11	3.10	.1226
8	Participants, n = 23	3.14	2.81–3.46	3.20	.7570
	Iterations, n = 1001	3.14	3.13–3.14	3.14	.1505
	Iterations, n = 2001	3.14	3.13–3.14	3.14	.1569
9	Participants, n = 23	3.18	2.85–3.52	3.25	.7770
	Iterations, n = 1001	3.18	3.17–3.19	3.18	.1576
	Iterations, n = 2001	3.18	3.18–3.19	3.19	.1580
10	Participants, n = 23	3.17	2.93–3.40	3.18	.5436
	Iterations, n = 1001	3.17	2.95–3.38	3.16	.1088
	Iterations, n = 2001	3.17	2.95–3.38	3.15	.1098
11	Participants, n = 23	3.30	2.95–3.66	3.39	.8220
	Iterations, n = 1001	3.30	3.29–3.31	3.30	.1685
	Iterations, n = 2001	3.31	3.30–3.31	3.31	.1652
12	Participants, n = 23	3.09	2.74–3.43	3.14	.7930
	Iterations, n = 1001	3.09	3.08–3.10	3.09	.1600
	Iterations, n = 2001	3.09	3.08–3.10	3.09	.1583
13	Participants, n = 23	2.87	2.54–3.20	2.90	.7570
	Iterations, n = 1001	2.86	2.86–2.87	2.87	.1489
	Iterations, n = 2001	2.87	2.86–2.88	2.87	.1528
14	Participants, n = 23	2.53	2.23–2.82	2.59	.6821
	Iterations, n = 1001	2.52	2.52–2.53	2.53	.1412
	Iterations, n = 2001	2.83	2.82–2.83	2.83	.1578
15	Participants, n = 23	2.83	2.49–3.16	2.86	.7780
	Iterations, n = 1001	2.82	2.81–2.83	2.83	.1594
	Iterations, n = 2001	2.83	2.82–2.83	2.83	.1578
16	Participants, n = 23	3.23	2.91–3.54	3.25	.7340
	Iterations, n = 1001	3.23	3.22–3.24	3.23	.1495
	Iterations, n = 2001	3.23	3.22–3.23	3.23	.1490
17	Participants, n = 23	3.35	3.01–3.68	3.39	.7750
	Iterations, n = 1001	3.35	3.34–3.36	3.35	.1569
	Iterations, n = 2001	3.35	3.34–3.36	3.35	.1557
18	Participants, n = 23	3.39	3.08–3.70	3.43	.7220
	Iterations, n = 1001	3.39	3.39–3.40	3.40	.1455
	Iterations, n = 2001	3.39	3.38–3.40	3.39	.1483
19	Participants, n = 23	3.33	3.01–3.65	3.42	.7390
	Iterations, n = 1001	3.33	3.32–3.33	3.33	.1536
	Iterations, n = 2001	3.33	3.33–3.34	3.33	.1528
20	Participants, n = 23	3.57	3.28–3.85	3.63	.6620
	Iterations, n = 1001	3.57	3.56–3.58	3.57	.1318
	Iterations, n = 2001	3.56	3.56–3.57	3.56	.1355
21	Participants, n = 23	2.96	2.65–3.26	3.00	.7057
	Iterations, n = 1001	2.96	2.67–3.24	2.93	.1427
	Iterations, n = 2001	2.96	2.68–3.23	2.95	.1384
22	Participants, n = 23	3.39	3.11–3.68	3.43	.6560
	Iterations, n = 1001	3.39	3.38–3.40	3.39	.1385
	Iterations, n = 2001	3.39	3.38–3.39	3.39	.1323
23	Participants, n = 23	3.30	2.95–3.66	3.39	.8220
	Iterations, n = 1001	3.30	3.29–3.31	3.30	.1706
	Iterations, n = 2001	3.30	3.30–3.31	3.31	.1714
24	Participants, n = 23	3.39	3.18–3.61	3.38	.4990
	Iterations, n = 1001	3.39	3.39–3.40	3.39	.1024
	Iterations, n = 2001	3.39	3.39–3.40	3.39	.0986
25	Participants, n = 23	3.48	3.22–3.73	3.52	.5930
	Iterations, n = 1001	3.48	3.47–3.49	3.48	.1243
	Iterations, n = 2001	3.47	3.47–3.48	3.48	.1195
26	Participants, n = 23	2.83	2.42–3.23	2.86	.9370
	Iterations, n = 1001	2.83	2.81–2.84	2.83	.1874
	Iterations, n = 2001	2.82	2.82–2.83	2.82	.1915
27	Participants, n = 23	2.89	2.61–3.18	2.93	.6670
	Iterations, n = 1001	2.89	2.89–2.90	2.90	.1387
	Iterations, n = 2001	2.89	2.89–2.90	2.89	.1369
28	Participants, n = 23	3.26	2.91–3.61	3.34	.8100
	Iterations, n = 1001	3.26	3.25–3.27	3.27	.1744
	Iterations, n = 2001	3.26	3.25–3.26	3.26	.1675
29	Participants, n = 23	2.83	2.44–3.21	2.86	.8870
	Iterations, n = 1001	2.82	2.81–2.84	2.83	.1845
	Iterations, n = 2001	2.83	2.82–2.84	2.83	.1814
30	Participants, n = 23	3.43	3.12–3.75	3.48	.7280
	Iterations, n = 1001	3.44	3.43–3.45	3.44	.1496
	Iterations, n = 2001	3.44	3.43–3.44	3.44	.1473
31	Participants, n = 23	3.09	2.74–3.43	3.14	.7930
	Iterations, n = 1001	3.08	3.07–3.09	3.09	.1576
	Iterations, n = 2001	3.09	3.08–3.09	3.09	.1639
32	Participants, n = 23	3.06	2.75–3.36	3.11	.7050
	Iterations, n = 1001	3.05	3.04–3.06	3.05	.1500
	Iterations, n = 2001	3.05	3.05–3.06	3.06	.1428
33	Participants, n = 23	3.17	2.84–3.51	3.24	.7780
	Iterations, n = 1001	3.17	3.16–3.18	3.17	.1602
	Iterations, n = 2001	3.17	3.17–3.18	3.18	.1579
34	Participants, n = 23	3.26	2.94–3.59	3.34	.7520
	Iterations, n = 1001	3.27	3.26–3.28	3.28	.1528
	Iterations, n = 2001	3.26	3.26–3.27	3.27	.1512
35	Participants, n = 23	3.39	3.11–3.68	3.43	.6560
	Iterations, n = 1001	3.39	3.39–3.40	3.40	.1333
	Iterations, n = 2001	3.39	3.38–3.39	3.39	.1314
36	Participants, n = 23	3.06	2.85–3.26	3.06	.4740
	Iterations, n = 1001	3.06	3.05–3.07	3.06	.0991
	Iterations, n = 2001	3.06	3.05–3.06	3.06	.0972
37	Participants, n = 23	3.32	3.05–3.59	3.35	.6310
	Iterations, n = 1001	3.31	3.30–3.32	3.31	.1282
	Iterations, n = 2001	3.31	3.31–3.32	3.31	.1294
38	Participants, n = 23	2.89	2.60–3.18	2.93	.6670
	Iterations, n = 1001	2.89	2.88–2.90	2.89	.1369
	Iterations, n = 2001	2.89	2.89–2.90	2.89	.1346
39	Participants, n = 23	3.22	2.90–3.54	3.24	.7360
	Iterations, n = 1001	3.22	3.21–3.23	3.22	.1543
	Iterations, n = 2001	3.48	3.47–3.48	3.48	.1022
40	Participants, n = 23	3.48	3.16–3.79	3.53	.7300
	Iterations, n = 1001	3.48	3.47–3.49	3.48	.1519
	Iterations, n = 2001	3.48	3.47–3.48	3.48	.1465
41	Participants, n = 23	3.39	3.08–3.70	3.43	.7220
	Iterations, n = 1001	3.40	3.39–3.41	3.40	.1492
	Iterations, n = 2001	3.40	3.39–3.40	3.40	.1450
42	Participants, n = 23	3.65	3.44–3.86	3.67	.4870
	Iterations, n = 1001	3.65	3.64–3.66	3.65	.1041
	Iterations, n = 2001	3.65	3.65–3.66	3.65	.1003
43	Participants, n = 23	3.61	3.32–3.89	3.68	.6560
	Iterations, n = 1001	3.61	3.60–3.62	3.61	.1338
	Iterations, n = 2001	3.61	3.60–3.61	3.61	.1362
44	Participants, n = 23	3.61	3.39–3.82	3.62	.4990
	Iterations, n = 1001	3.61	3.60–3.62	3.61	.1020
	Iterations, n = 2001	3.61	3.60–3.61	3.61	.1036
45	Participants, n = 23	3.30	3.00–3.61	3.34	.7030
	Iterations, n = 1001	3.31	3.30–3.32	3.31	.1461
	Iterations, n = 2001	3.30	3.30–3.31	3.30	.1424
46	Participants, n = 23	3.43	3.07–3.80	3.53	.8430
	Iterations, n = 1001	3.43	3.42–3.45	3.44	.1814
	Iterations, n = 2001	3.44	3.43–3.44	3.44	.1735
47	Participants, n = 23	3.39	3.11–3.68	3.43	.6560
	Iterations, n = 1001	3.39	3.38–3.40	3.39	.1306
	Iterations, n = 2001	3.39	3.38–3.40	3.39	.1368
48	Participants, n = 23	3.48	3.26–3.70	3.48	.5110
	Iterations, n = 1001	3.48	3.47–3.48	3.48	.1037
	Iterations, n = 2001	3.48	3.47–3.48	3.48	.1022
49	Participants, n = 23	3.61	3.39–3.82	3.62	.4990
	Iterations, n = 1001	3.61	3.60–3.62	3.61	.1018
	Iterations, n = 2001	3.60	3.60–3.61	3.60	.1003
50	Participants, n = 23	3.26	2.94–3.59	3.29	.7520
	Iterations, n = 1001	3.25	3.25–3.26	3.26	.1490
	Iterations, n = 2001	3.27	3.26–3.27	3.27	.1527
51	Participants, n = 23	3.30	3.03–3.58	3.34	.6350
	Iterations, n = 1001	3.31	3.30–3.31	3.31	.1313
	Iterations, n = 2001	3.30	3.30–3.31	3.30	.1316
52	Participants, n = 23	3.39	3.11–3.68	3.43	.6560
	Iterations, n = 1001	3.39	3.38–3.40	3.39	.1336
	Iterations, n = 2001	3.39	3.39–3.40	3.39	.1325
53	Participants, n = 23	3.18	2.85–3.50	3.25	.7530
	Iterations, n = 1001	3.18	3.17–3.19	3.18	.1542
	Iterations, n = 2001	3.18	3.17–3.19	3.18	.1543
54	Participants, n = 23	3.18	2.85–3.50	3.25	.7530
	Iterations, n = 1001	3.17	3.16–3.18	3.17	.1556
	Iterations, n = 2001	3.18	3.18–3.19	3.18	.1550

In general, the means of the expert scores per critical process remained stable across the three data sets (one original and two augmented); the confidence intervals of the three samples were overlapping for each of the critical processes with the confidence intervals in bigger samples being more compact; the trimmed mean exhibited stability, and the standard deviation decreased with increasing number of experts. When the standard deviation decreases, this indicates that the typical deviation of expert opinions has not increased relative to the increased number of responses. Therefore, we can conclude that the original expert sample yielded results concerning the importance of the critical processes in the patient safety tool that were comparable to the results of the expanded samples.

The Delphi survey results showed stability after bootstrap resampling data expansion. Therefore, *the stability of the results of the bootstrap data expansion validated the patient safety strategic planning tool developed by the Delphi study on patient safety *[29].

## Discussion

### Application of Delphi studies in healthcare

In the field of healthcare, the Delphi method has been used in planning for the future and formulating policies and programs in biomedical research, behavioral research, mental health, reproductive health, pharmacology, services for the elderly, family planning services, accidents and injuries, development of core competencies for advanced nursing practitioners and development of clinical care protocols [27,36-41]. The Delphi method, as a useful way of identifying and measuring uncertainty, has been widely utilized in medical and health services research to explore issues in health services organizations, to support design of educational programs, to define professionals' roles, to define effects of medical staffing levels, to develop criteria for appropriateness of clinical treatment, and to make long-term projections of need for patient care [42]. The Delphi methodology also has been used to modify the National Board of Medical Examiners (NBME) Medicine Subject Exam (Shelf) in order to align the national exams with the internal medicine clerkship curriculum developed by the Society of General Internal Medicine (SGIM) and the Clerkship Directors in Internal Medicine (CDIM) [43]. Consensus building through Delphi survey technique can contribute significantly to broadening knowledge and effective decision making in health and social care [22]. Furthermore, the Delphi approach can be used as a senior management education tool, environmental planning tool, and for comparison with similar healthcare institutions. Putting together the structure of a model, developmental planning and exploration of policy options are among the explicit application areas identified since the early 1970's [26]. Additionally, the Delphi method has been used to delineate the barriers to performance in health services and identify three types of barriers to optimal healthcare performance: solution development barriers, problem selection barriers, and evaluation barriers. It has been argued that the forecasting accuracy of Delphi studies is strongly reliable; for example, a Delphi study with medical doctors evaluating the forecasting application of the method, revealed that in 75% of the cases the estimated values proved to be less than 10% different from the observed [26].

### Challenges in selection of a Delphi panel

The questions arising around the formation of a Delphi panel are typical for selection and formation of any group – committee, task force, panel, or study group. Thus, while panel member selection is a problem that should be addressed, it is by no means unique to Delphi studies. It has been argued that the amount of bias expressed by study participants is offset by the fact that in answering the questions each participant exhibits a standard deviation which is comparable to, or greater than participant's individual mean (i.e., an optimistic panellist is pessimistic in some of his/her responses, and vice versa) [26].

The selection of criteria that would qualify an individual to participate on the Delphi panel depends on the context, scope and aims of the particular study. Some of the general criteria include:

• Knowledge and practical engagement with the issue under investigation

• Capacity and willingness to contribute to the exploration of a particular problem

• Assurance that sufficient time will be dedicated to the Delphi exercise,

• Good written communication skills

• Experts' skills and knowledge need not necessarily be accompanied by standard academic qualifications or degrees [44].

Delphi participants are purposefully selected to apply their knowledge and experience to a certain issue based on a criteria set. For example, the experts for a national two-round Delphi study on the effectiveness and risks of coronary angiography were chosen on the basis of their clinical expertise, community influence, and diversity of geographic location [45]. Since the Delphi method relies on repeated questionnaires to the same initially selected sample of participants, the method requires a continued commitment from the panellists and is heavily dependant on the time and continued involvement on the part of the study participants. The widespread employment of electronic communications calls for consideration of the computer literacy and skills of the target sample before utilizing electronic means of communication [22]. Since the sample size for Delphi panels has not been established, it is important to know whether the selected Delphi panel for a particular study would yield stable results.

### Utilization of bootstrap in healthcare research

During the last decade, the use of bootstrap data expansion in healthcare research became more prominent. Some examples of utilization of bootstrap in contemporary healthcare research include: constructing confidence intervals for treatment differences, analysing cost-effectiveness in randomized controlled trials, assessing provider performance for providers with small numbers of observed events, and studying the genetic linkages in viral genome sequences [46-51]. There is a growing validation of the value of bootstrap in medical statistics and an increasing recognition that the bootstrap technique can supplement and extend the conventional statistical thinking. Bootstrap can be used for calculation of confidence intervals, hypothesis testing, linear regression and correlation in variable prediction, and non-linear regression in immunoassay techniques [33]. The bootstrap method, with its computational simplicity and performance similar to the fully Bayesian approaches, was found to be a very useful addition to healthcare researchers' statistical toolkit [52].

## Conclusion

Although experiments carried out in the 1950's and 1960's suggested that group error is reduced with increased group size, the sample size for constructing a Delphi panel is not a statistically-bound decision and good results can be obtained by a comparatively small group of homogenous experts [44]. However, the size of a "small" Delphi sample has not been unequivocally established.

The findings of this study are important because:

1. It was found that reliable outcomes could be obtained with a Delphi panel consisting of a relatively small number of Delphi experts (23) selected via strict inclusion criteria. This finding is particularly important for conducting Delphi surveys in knowledge or practice fields where the population of experts (the total number of qualifying knowledgeable individuals) is limited. ***Experts who have similar training and general understanding in the field of interest allow for effective and reliable utilization of a small sample from a limited number of experts in the field of study.***

2. The stability of the results from a Delphi survey conducted with a small number of experts from a defined field of study was established. It is hypothesized that Delphi surveys in other fields of study, conducted with a small number of experts with similar training and knowledge, would also yield reliable results. Given the fact that the number of specialized experts in a particular field may be limited, this study validated the stability of response characteristics of a small expert sample from limited numbers of experts. ***Therefore, consistency of expert training may allow utilization of small numbers of experts in fields where many experts may be available but participation of a limited number of experts on the Delphi panel may be more practical.***

## Competing interests

The authors declare that they have no competing interests.

## Authors' contributions

RBA, HT and BRC developed the project idea and the study design. RBA collected the data. RBA and HT conducted the study. HT provided statistical advice and BRC provided conceptual advice. All authors contributed to the analysis of data. The manuscript was initially drafted by RBA. HT and BRC critically reviewed and edited the manuscript. All authors contributed to and approved the final version submitted for publication.

## Pre-publication history

The pre-publication history for this paper can be accessed here:


